# Supplementary material for: Wearable and Washable MnO2−Zn Battery Packaged by Vacuum Sealing
Source: Nanomaterials (Basel). 2023 Jan 7;13(2):265. doi: 10.3390/nano13020265 (PMC9864546; doi:10.3390/nano13020265)
Supplement: Supplementary file 1 [file nanomaterials-13-00265-s001.zip › nanomaterials-2141817-supplementary.pdf]

Supplementary Information

Wearable and Washable MnO_2–Zn Battery Packaged by Vacuum Sealing

Jun Ho Noh [1,2,†], Myoungeun Oh [1,†], Sunjin Kang [1], Hyeong Seok Lee [1], Yeong Jun Hong [1], Chaeyeon Park [1], Raeyun Lee [1] and Changsoon Choi [1,3,*]

[1] Department of Energy and Materials Engineering, Dongguk University, 30 Pildong−ro, 1−gil, Jung−gu, Seoul 04620, Republic of Korea
[2] Department of Advanced Battery Convergence Engineering, Dongguk University, 30 Pildong−ro, 1−gil, Jung−gu, Seoul 04620, Republic of Korea
[3] Research Center, Sillo Incorporation, 30 Pildong−ro, 1−gil, Jung−gu, Seoul 04620, Republic of Korea
* Correspondence: cschoi84@dongguk.edu
† These authors contributed equally to this work.

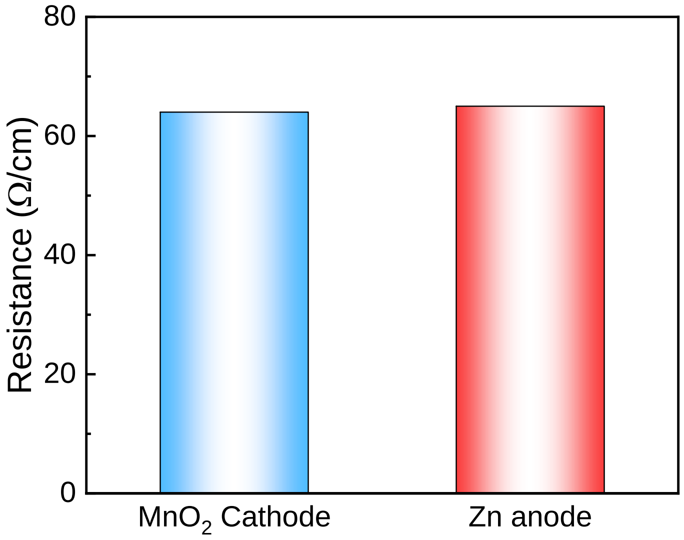

Figure S1. Electrical resistances of the MnO_2 yarn cathode and Zn yarn anode.

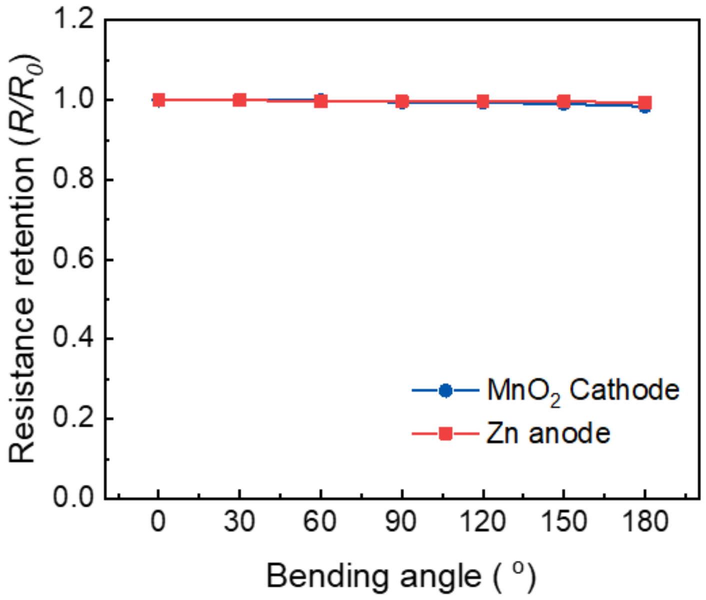

Figure S2. Resistance retention ratios (R/R_0) of the MnO_2 yarn cathode and Zn yarn anode under various bending angles from 0° to 180°.

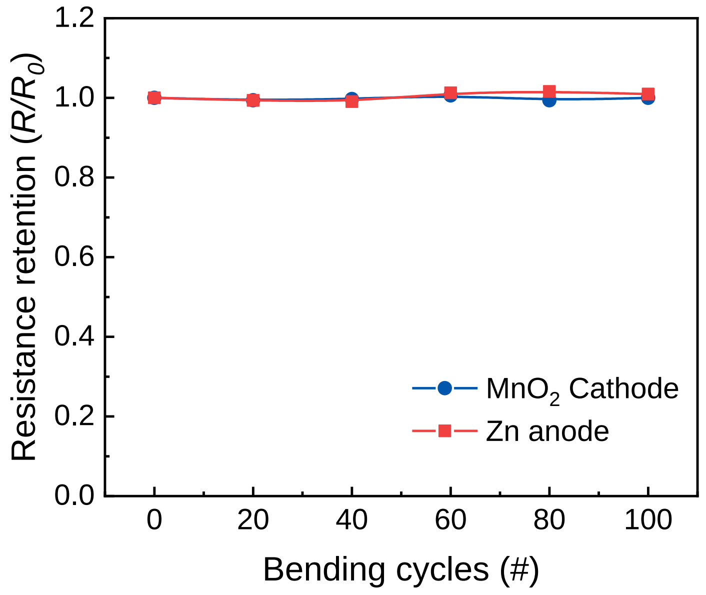

Figure S3. Resistance retention ratios (R/R_0) of the MnO_2 yarn cathode and Zn yarn anode after 100 cycles with a bending angle of 180°.

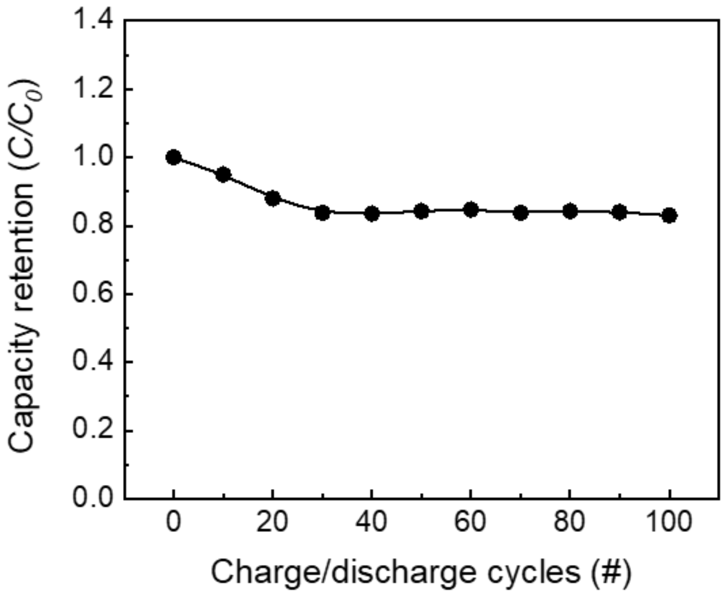

Figure S4. Capacity retention ratios (C/C_0) of the MnO_2−Zn textile battery during 100 charge/discharge cycles.
